# Turnover of missense mutant cytosolic proteins proceeds in the absence of major quality control E3 ligases

**DOI:** 10.17912/micropub.biology.001990

**Published:** 2026-03-27

**Authors:** Heather A. Baker, Claire Grall-Johnson, Paulina M. Budzyńska, John Kim, Thibault Mayor

**Affiliations:** 1 Biochemistry and Molecular Biology, University of British Columbia, Vancouver, BC, CA; 2 Michael Smith Laboratories, University of British Columbia, Vancouver, BC, CA; 3 Edwin S.H. Leong Centre for Healthy Aging, University of British Columbia, Vancouver, BC, CA

## Abstract

Protein quality control (QC) safeguards cellular proteostasis by directing misfolded proteins for degradation via the ubiquitin-proteasome system. QC is compartmentalized within cells, and the key proteins involved in the turnover of cytosolic proteins with mutations in mammalian cells are not well defined. Using a fluorescent reporter assay that provides a readout for protein stability, we examined the contributions of known QC E3 ligases (STUB1, UBE2O, UBR4, UBR5, and HUWE1) on the turnover of disease-associated missense variants. Loss of individual ligases did not consistently stabilize substrates, indicating that none of these E3s appear to broadly recognize missense mutant proteins.

**Figure 1. Individual deletion of the candidate E3 ligases STUB1, UBR4, UBR5, HUWE1, or UBE2O has minimal impact on the turnover of cytosolic missense mutant substrates:** A) Predominant subcellular localization of the QC E3 ligases and reporters used in this study. Dark and light blue represent strong or circumstantial localization evidence in the indicated compartment, respectively, while white indicates no evidence. Data for subcellular localization of E3 ligases was determined through Human Protein Atlas, Uniprot annotations, and existing literature. STUB1, UBE2O, and UBR4 have been reported to localize to both the cytosol and nucleus, whereas UBR5 and HUWE1 are primarily nuclear (Go et al., 2021; Kim et al., 2018; Mashtalir et al., 2014; Tasaki et al., 2013). Subcellular localization of the reporters was determined by microscopy in a previous study (Baker et al., 2025). B) Western blots of indicated E3 ligase KO cells along with the parental HEK293T cells (HEK). * = indicates unspecific band. HUWE1 KO clones were a gift from Yihong Ye’s lab (Xu et al., 2016). C) Schematic of bicistronic fluorescent reporter construct used in D). D) Log _2_ (EGFP/DsRed) levels of the missense mutant reporters and corresponding WT in STUB1 KO cells. Three independent KO clones of STUB1 were used. Results from a one-way ANOVA test are shown with the mean and standard deviation (n=3-9, p > 0.05 = ns). E) Schematic of bicistronic fluorescent reporter used in F-J. F) Log _2_ (mClover2/RFP) of the mutant reporter and corresponding WT in HEK293T cells. Results from an unpaired t-test are shown with the mean and standard deviation (n=3; **** p < 0.0001). G-I) Log _2_ (mClover2/RFP) of the missense mutant reporters CBS L456P, GNMT H177N, and TPMT A80P expressed in (G) UBR4, (H) UBR5 and (I) HUWE1 WT and KO clone(s). Results from an unpaired t-test are shown with the mean and standard deviation (n=3; p > 0.05 = ns, * p < 0.05, ** p <0.01, *** p < 0.001). J) Density plot of the Log _2_ (mClover2/RFP) signal of CBS L456P, GNMT H177N, and TPMT A80P in UBE2O WT and KO clones (n=1).

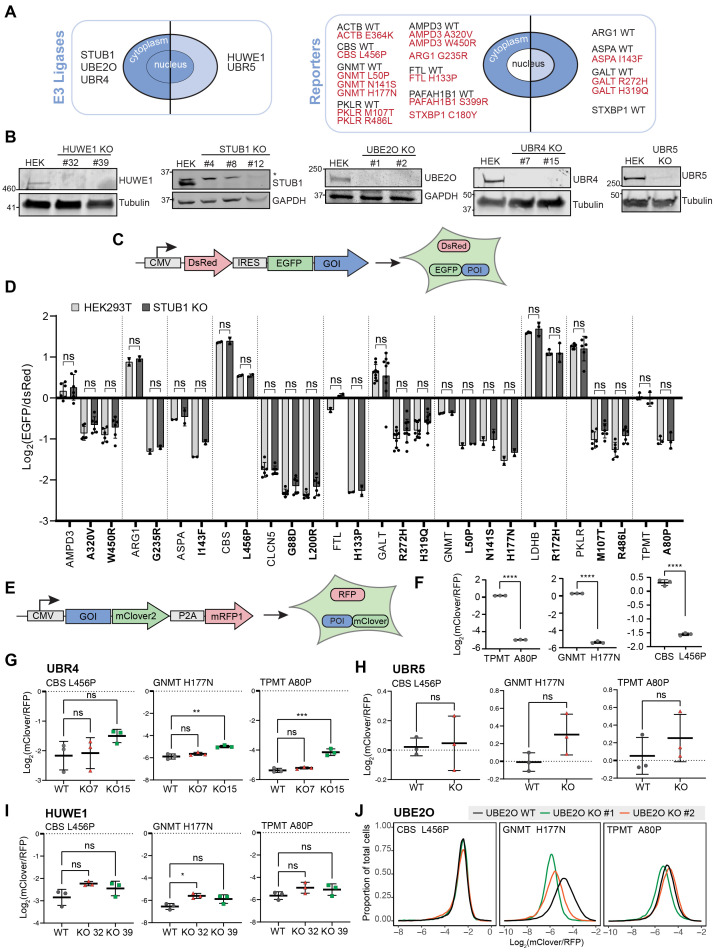



## Description

Proteostasis (protein homeostasis) relies on coordinated pathways that control protein synthesis, folding, and degradation. Even under optimal conditions, protein folding can be challenging due to high energy barriers, especially for proteins with complex or larger structures (Balchin et al., 2016). When folding fails, misfolded substrates are typically targeted for degradation, primarily through the ubiquitin-proteasome system (UPS). Disruption of these processes causes proteotoxic stress and contributes to diseases, including neurodegeneration, cancer, and hereditary misfolding disorders (Baker & Bernardini, 2021; Yerbury et al., 2016).

UPS-mediated degradation is a major pathway conserved among eukaryotes (Komander & Rape, 2012) that relies on E1, E2, and E3 enzymes to coordinate the transfer of ubiquitin to substrate proteins for proteasomal degradation. Over 600 E3s are predicted to exist in mammals (Liu et al., 2019), including several ubiquitin ligases that selectively target misfolded or aberrant proteins for degradation in different cellular compartments. For example, the endoplasmic reticulum–associated degradation (ERAD) pathway targets misfolded secretory or membrane proteins for proteasomal degradation via the E3 ligases HRD1, AMFR, and MARCH6/TEB4 (Olzmann et al., 2013). Much of our understanding of ERAD has come from studies of model substrates such as mutant CFTR, which revealed the network of chaperones and E3s that collaborate in ERAD (Christianson et al., 2023). In contrast, QC in other compartments is less well-characterized.

In the cytosol—the major site of protein synthesis—the identities and functions of QC E3s remain incompletely defined. Several mammalian E3 ligases with well-established roles in protein QC have been implicated in pathways to safeguard proteostasis. STUB1/CHIP was the first cytosolic QC E3 to be characterized, cooperating with the chaperones Hsp70/Hsp90 to ubiquitinate misfolded proteins and promote their degradation (Connell et al., 2001; Murata et al., 2001). More recently, HUWE1, the hybrid E2/E3 UBE2O, UBR5, UBR4 (in complex with KCMF1), HERC1, and HERC2 have been shown to ubiquitinate orphan proteins and unassembled subunits (Carrillo Roas et al., 2025; Mark et al., 2023; Nguyen et al., 2017; Xu et al., 2016; Yagita et al., 2023; Yanagitani et al., 2017; Zavodszky et al., 2021). UBR4 and UBR5 have also been shown to cooperatively target misfolded proteins for degradation shortly following translation (Yau et al., 2017). Whereas these E3 ligases reside, at least in part, in the cytosol ( Figure 1A ), their contribution to targeting disease-associated missense variants of cytosolic proteins remains largely unexplored.

Our lab previously established a series of fluorescent reporters corresponding to unstable, disease-associated mutant variants to monitor cytosolic QC (Baker et al., 2025). These reporters were shown by fluorescence microscopy in our study to be predominantly cytosolic, with limited nuclear signal in some cases ( Figure 1A ). Using this system, we tested whether candidate E3 ligases contribute to the turnover of these cytosolic substrates.

To investigate whether these E3 ligases contribute to cytosolic reporter turnover, we generated knockout (KO) HEK293T cell lines for STUB1, UBE2O, UBR4, and UBR5, and obtained HUWE1 KO cells from the Ye lab (Xu et al., 2016). These ligases were selected based on previous links to protein QC. Knockouts were generated using a dual-nickase CRISPR strategy with &nbsp;two guide RNAs (sgRNAs) targeting early exons, selected by CHOPCHOPv3 (Labun et al., 2019) (see Reagents Table). Single-cell clones were expanded and validated by Western blotting ( Figure 1B ), confirming at least one validated KO clone per ligase for use in downstream assays.

We first assessed the extent of STUB1’s role in cytosolic protein QC, since it was originally proposed to be a major E3 ligase targeting misfolded proteins. To do so, we used dual-fluorescent DsRed-EGFP reporters containing cytosolic missense mutants and their corresponding wild-type (WT) proteins (Baker et al., 2025). DsRed is translated first, followed by an internal ribosome entry site (IRES) driving translation of the EGFP-tagged cytosolic protein of interest from the same mRNA ( Figure 1C ). In this system, unstable mutant proteins lead to low EGFP fluorescence relative to DsRed, and loss of a turnover-promoting E3 ligase should result in reporter stabilization and an increase in the normalized EGFP signal. We measured reporter stability of 17 unstable mutants in 11 genes across three independent STUB1 KO clones. Consistent with our previous study, all mutants showed a decrease in the normalized fluorescence relative to their WT in the control cells ( Figure 1D ), indicative of degradation. Surprisingly, loss of STUB1 had no significant impact on the stability of any mutant reporters. These data suggest that STUB1 does not play a dominant role in regulating the turnover of this wide panel of cytosolic unstable mutant reporters.

We next examined additional E3 ligases previously linked to cytosolic QC to determine whether any of them may have a more dominant role. Here, we use an alternative strategy with stably expressed reporters. In this system, a subset of the cytosolic missense mutants is fused N-terminally to mClover2, followed by a P2A cleavage site and the RFP to reduce variability during transfection ( Figure 1E ). Similar to our previous construct, both RFP and the mClover2 fusion protein are translated from a single transcript, allowing RFP to serve as an internal control for expression while mClover2 reports on the stability of the protein of interest. We focused on three disease-associated enzyme variants—cystathionine beta-synthase (CBS) L456P, glycine N-methyltransferase (GNMT) H177N, and thiopurine S-methyltransferase (TPMT) A80P. The three missense mutants expressed in HEK293T cells were present at lower levels compared to the wild-type proteins, indicating that reporter turnover is not impaired by fluorophore positioning ( Figure 1F ). These reporters were introduced into UBR4, UBR5, HUWE1, and UBE2O KO cells using lentiviral transduction. In all of the tested KO E3s, we did not observe any coherent reporter stabilization ( Figure 1G- J). In UBR4 KOs, a modest but significant stabilization–as indicated by an increase in the log _2_ (mClover2/RFP) ratio–was observed in clone #15 but not in clone #7 ( Figure 1G ). UBR5 KO showed no significant effect on any of the mutants tested, despite a marginal signal increase ( Figure 1H ). Similarly, HUWE1 KO did not significantly stabilize any reporter, with the exception of a modest increase in the GNMT H177N reporter levels in clone #32 ( Figure 1I ). Intriguingly, UBE2O KOs had a different effect with a destabilization of GNMT H177N ( Figure 1J ), although this analysis was not repeated. Altogether, these results indicate that none of the tested E3s had a dominant role in the degradation of the unstable mutant proteins examined.

In this study, we set out to examine the role of several E3 ligases in the turnover of unstable cytosolic protein variants using fluorescent reporter assays. None of these ligases (STUB1, UBE2O, UBR4, UBR5, and HUWE1) led to a consistent effect on reporter stabilization when knocked out ( Figure 1D, G-J). Importantly, the modest effects occasionally observed for UBR4 and HUWE1 were inconsistent across clones ( Figure 1G, I). Together, these results indicate that the tested ligases are not the primary mediators of turnover for these substrates.

One possible explanation is that these E3s are functionally redundant. In yeast, for example, the E3 ligases Ubr1 and San1 have been shown to recognize and ubiquitinate the same substrates (Heck et al., 2010). Only simultaneous deletions of Ubr1 and San1 have also been shown to stabilize protein QC substrates to the same extent as proteasome inhibition (Samant et al., 2018). Mammalian cells may have analogous E3 ligase redundancies, potentially masking their individual roles in cytosolic QC. To address this, future studies should test the effects of combinatorial E3 ligase knockouts.

A further limitation of this work is the generation and characterization of KO lines, which can be complicated by unintended byproducts or selective genetic pressures (Wang et al., 2024). Despite the generation of a homozygous deletion, residual expression of unexpected, truncated protein products could persist. For example, the presence of residual protein expression was observed by mass spectrometry for approximately one-third of genetically verified deletions in HAP1 cell lines (Smits et al., 2019). This is particularly relevant for HUWE1 and UBR4, which are exceptionally large proteins (482 kDa and 570 kDa, respectively) and thus have an increased chance of generating truncated or reinitiated products that retain partial functionality. However, we did not detect truncated forms using C-terminal-targeting antibodies of each ligase, making it unlikely that truncated or splice variants lacking the epitope would escape detection. Another possibility is that compensatory pathways become upregulated during KO cell expansion, enabling the cells to re-establish rapid turnover of the unstable mutant proteins. Nonetheless, RNAi-mediated depletion of STUB1 was also insufficient to affect the turnover of several assessed mutants (Sahil Chandhok, personal communication), indicating that this E3 ligase does not play a dominant role in protein QC in the cells tested. More work is needed to determine which E3 ligases—individually or in combination—mediate the degradation of these unstable mutant proteins.

## Methods

**Cell culture, transfections, and virus generation**

HEK293T cells (ATCC) were cultured in DMEM, high glucose (Gibco) supplemented with 10% FBS (Gibco) + 1% penicillin-streptomycin (Gibco) and maintained in a humidified incubator at 37 °C with 5% CO₂. Transient transfections of missense mutant and WT proteins cloned into the MSCV-CMV-DsRed-IRES-EGFP-DEST reporter plasmid (gift from Stephen Elledge; Addgene plasmid #41941 (Yen et al., 2008)) were performed using FuGENE 6 (Promega) according to the manufacturer’s instructions. For lentivirus production, HEK293T cells were co-transfected with psPAX2 (gift from Didier Trono; Addgene plasmid #12260), pMD2.G (gift from Didier Trono; Addgene plasmid #12259), and the transfer plasmid pLenti6.2-ccdB-mClover2-P2A-mRFP1 (gift from Mikko Taipale, University of Toronto; unpublished) expressing the mutant or WT protein of interest. Media was replaced 24 hours after transfection. Viral supernatants were harvested at 48 hours, clarified using a 0.45 mm filter, and used to transduce target cells in the presence of 4 μg/ml of polybrene (EMD-Millipore). Transduced cells were expanded and selected with 6 μg/ml of blasticidin (Invitrogen) to establish stable lines.

&nbsp;

**Flow cytometry**

To assess transfected reporters, cells were collected 48 hours after transfection. To assess cell lines stably expressing the reporters, cells were collected 24 hours after seeding. Cells were resuspended in 1X PBS + 2% FBS and analyzed on the CytoFlex (Beckman Coulter). Cells were first gated on forward scatter (FSC) versus side scatter (SSC) to exclude debris, followed by FSC-A vs FSC-W to exclude doublets. Cells positively transfected with the reporter were identified by events > 10 ^4^ arbitrary fluorescence units (a.u.) in the ECD-A channel.

&nbsp;

**Generation of CRISPR/Cas9 KO cells**

CRISPR/Cas9 knockout cell lines were generated in HEK293T cells. Guide RNAs targeting the E3 ligases were designed using CHOPCHOPv3 (see Reagents Table) and cloned into the pU6_sgRNA_CBh_Cas9_PGK_Venus vector (gift from Sheila Teves, University of British Columbia). Cells were transfected using FuGENE 6 (Promega) and 24 hours after transfection, cells were collected, and single-cell clones were isolated by fluorescence-activated cell sorting (FACS) on the Influx (BD Biosciences) for Venus+ clones. Clones were sorted into a 96-well plate in media composed of 50% fresh complete DMEM + 30% conditioned DMEM (medium collected from parental cells at 50% confluency) + 20% FBS. Homozygous knockouts were confirmed by PCR of the target locus, and loss of protein expression was validated by Western blotting.

&nbsp;

**Western blotting**

Cells were lysed in 1X RIPA buffer (50 mM Tris-HCl pH 7.5, 150 mM NaCl, 1% NP-40, 0.5% sodium deoxycholate, 0.1% SDS, 1 mM PMSF) supplemented with cOmplete, EDTA-free Protease Inhibitor Cocktail (Roche). Cells were incubated on ice for 30 minutes after the addition of lysis buffer then lysed using a water bath sonicator with ice water for 30s on, 30s off 2x on high. Cell lysate was then centrifuged at high speed for 20 min at 4°C. Protein supernatant was collected, and protein concentrations were determined using the BCA assay. Proteins were resolved by 7.5% Mini-PROTEAN TGX™ Precast Protein Gels (Bio-Rad). For UBE2O and STUB1, proteins were transferred to a 0.45 mm membrane using the TransBlot Turbo Transfer System (30 min at 25V) in 1X Towbin Buffer with 20% methanol. For UBR4, UBR5 and HUWE1, proteins were transferred to a 0.45 mm PVDF membrane (pre-activated in 100% methanol for 10s) using a Trans-Blot Plus Cell (Bio-Rad) at 20 V for 16h at 4°C in 1X Towbin Buffer with 10% methanol and 0.05% Tween-20. Membranes were blocked with 5% skim milk powder in 1X TBS with 0.1% Tween-20. Antibodies were also diluted with 5% skim milk powder in 1X TBS with 0.1% Tween-20. Primary antibodies used: STUB1 (Cell Signalling 2080S, 1:1000 dilution), UBE2O (Millipore Sigma HPA023605 1:1000), UBR4 (abcam ab86738, 1:1000), UBR5 (Cell Signalling D608Z, 1:1000), HUWE1 (Bethyl A300-486A, 1:1000), g-Tubulin (Sigma T6557, 1:5000), GAPDH (UBC AbLab Mouse, 1:10,000). Secondary antibodies used: IRDye 680RD anti-mouse (Li-Cor, 1:10,000), IRDye 800CW anti-rabbit (Li-Cor, 1:10,000).&nbsp; Blots were imaged using the Odyssey DLx Imager (Li-Cor).

**&nbsp;**

**Data analysis**

Flow cytometry data was analyzed using FlowJo (10.10). All data was visualized and statistics were calculated using R (4.5.1), RStudio (2025.05.1+513), and GraphPad Prism (10.1.2).

## Reagents

**sgRNA sequences used for CRISPR–Cas9–mediated knockout**

**Table:** 

**Target Gene**	**sgRNA ID**	**sgRNA Sequence**
UBR4	sgUBR4-1	AGTCATCGAGAGGTACCGGG
UBR4	sgUBR4-2	CGACGGAAGATGGCGACGAG
UBR5	sgUBR5-1	CGGGTGAACCACGAAATGGA
UBR5	sgUBR5-2	TTCGTGGTTCACCCGCTGCC
STUB1	sgSTUB1-1	GCTGGACGGGCAGTCTGTGA
STUB1	sgSTUB1-2	GAATCGCGAAGAAGAAGCGC
UBE2O	sgUBE2O-1	CTGGTGTCGGGCCGTTACCG
UBE2O	sgUBE2O-2	CGTGCGCGTCCAGTGGTACC
